# Analysis of Pedestrian Street-Crossing Decision-Making Based on Vehicle Deceleration-Safety Gap

**DOI:** 10.3390/ijerph17249247

**Published:** 2020-12-10

**Authors:** Hongjia Zhang, Yingshi Guo, Yunxing Chen, Qinyu Sun, Chang Wang

**Affiliations:** School of Automobile, Chang’an University, Xi’an 710064, China; zhanghongjia@chd.edu.cn (H.Z.); guoyingshi_72@163.com (Y.G.); chenyunxing@chd.edu.cn (Y.C.); sunqingyu@chd.edu.cn (Q.S.)

**Keywords:** pedestrian, zebra crossings, decision-making model, vehicle deceleration, signal detection theory, autonomous vehicles

## Abstract

Numerous traffic crashes occur every year on zebra crossings in China. Pedestrians are vulnerable road users who are usually injured severely or fatally during human-vehicle collisions. The development of an effective pedestrian street-crossing decision-making model is essential to improving pedestrian street-crossing safety. For this purpose, this paper carried out a naturalistic field experiment to collect a large number of vehicle and pedestrian motion data. Through interviewed with many pedestrians, it is found that they pay more attention to whether the driver can safely brake the vehicle before reaching the zebra crossing. Therefore, this work established a novel decision-making model based on the vehicle deceleration-safety gap (VD-SGM). The deceleration threshold of VD-SGM was determined based on signal detection theory (SDT). To verify the performance of VD-SGM proposed in this work, the model was compared with the Raff model. The results show that the VD-SGM performs better and the false alarm rate is lower. The VD-SGM proposed in this work is of great significance to improve pedestrians’ safety. Meanwhile, the model can also increase the efficiency of autonomous vehicles.

## 1. Introduction

With the growth of urban vehicle ownership and the development of urban road facilities, pedestrian crashes are on the rise, and the issue of pedestrian safety has become increasingly serious. Among the traffic participants, pedestrians are most vulnerable to injury. According to the annual report on road traffic accident statistics released by the Traffic Administration Bureau of the Ministry of Public Security in China in 2017, the number of pedestrian injuries in traffic accidents was 35,058, accounting for 16.72% of all transportation modes. The fatalities were 17,286, accounting for 27.11% of all transportation modes [1].

The Chinese road traffic safety law stipulates that “when a vehicle passes a zebra crossing, it shall slow down; if a pedestrian is passing through the zebra crossing, the vehicle shall stop and yield to pedestrian.” In practice, few vehicles “actively” stop or slow down to yield to pedestrian on the zebra crossing.

The pedestrian traffic has the characteristics of discreteness and flexibility, which can cause serious conflict when meeting other traffic flows, thus affecting the normal operation of motor vehicles and leading to chaos at intersections. In order to improve pedestrian safety and traffic efficiency, it is necessary to analyze pedestrian street-crossing decision-making from the perspective of pedestrian psychology and behavior. The pedestrian decision-making model mainly includes machine learning-based and mathematical theory-based. Additionally, there are two kinds of pedestrian decision-making models based on mathematical theory, namely the critical safety gap model and the logistic regression model.

The critical safety gap was first developed as a method to calculate the capacity of unsignalized zebra crossings [2]. Raff et al. [3] defined the critical safety gap as a time headway. The number of rejected time headway is lower than the number of accepted time headway. Lobjois et al. [4] studied the relationship between the pedestrian street-crossing gap at different vehicle speeds and pointed out that the average acceptable gap decreased with an increase in vehicle speed. Lobjois et al. [5] studied the interaction between a pedestrian and a traffic flow to determine the relationship between the average pedestrian safety gap and traffic flow. Baltes et al. [6] analyzed the decision-making behavior of pedestrians at the unsignalized crosswalks and concluded that the traffic flow affects pedestrian street-crossing behavior. Lu et al. [7] studied the mean critical gap of the vehicle flow at a pedestrian street-crossing and obtained the normal distribution function of the acceptable crossing gap. The average critical gap is calculated by using the Raff method. Sun et al. [8] processed video data with tracking software, using a logit model to describe pedestrian behavior in choosing a safety gap at the signalized intersection crosswalks. Xiang et al. [9] determined the probability distribution of the pedestrian safety gap and constructed a pedestrian street-crossing safety level model. Based on multiple linear regressions and Harder’s method, Bai et al. [10] analyzed the time headway and the pedestrian street-crossing behavior factors at signalized intersections and obtained a critical gap of the pedestrian street-crossing. It was found that when the time headway is greater than 6.1 s, the Harder’s method can calculate the pedestrian critical gap more accurately.

Based on such factors as pedestrian gender, age, road condition, and the traffic environment, some scholars established a probability model of the pedestrian street-crossing safety gap to describe pedestrian decision-making [11,12,13]. Alver et al. [14] used a logit regression algorithm to model the pedestrian safety gap. The model takes into account pedestrian gender and age as well as the information about the surrounding vehicles. Ramesh et al. [15] established a pedestrian safety gap model that takes into account the influence of the road environment. Zhao et al. [16] established the probability model of the pedestrian safety gap by using a logistic regression algorithm. Taking into consideration the road factors, traffic environment, and pedestrians, the model can accurately reflect and predict the gap choice behavior of pedestrians. Zhu [17] established the mathematical relationship model between pedestrian street-crossing behavior probability and safety gap by using the measured data and verified the model. It was proved that the model has a good fitting degree and prediction accuracy, and compared with the existing logit model.

Pedestrian street-crossing models based on the acceptance gap, time to collision (TTC), and logistic regression are well known to scholars. Whether or not they perform well, from the perspective of mathematical theory, these methods provide a reasonable way to measuring pedestrian street-crossing. However, none of these approaches starts with what pedestrians are thinking when they cross the street. To which factors are pedestrians more sensitive when they cross the street? What are they more concerned about when crossing the street? Through interviewed with pedestrians, we found that most pedestrians consider the extent of the threat from the oncoming vehicle and think about whether the driver can safely brake the car without hitting them. It can be seen that vehicle deceleration is a well-known and accepted concept that concerns the public. Risto et al. [18], Petzoldt [19], and Zimmermann & Wettach [20] also pointed out that vehicle deceleration is an important indicator of whether pedestrians choose to cross the street. It can be used as an important clue for communication between vehicles and pedestrians. Olszewski et al. [21] pointed out that computational indicators, such as the safety gap and TTC are impractical for pedestrians. It is difficult for pedestrians to think about the current safety gap value or the current TTC value when crossing the street. These professional concepts are vague and unclear to pedestrians. Generally speaking, pedestrians crossing the street make mostly subjective judgments. Therefore, this work proposes a pedestrian street-crossing decision-making model based on the vehicle deceleration-safety gap (VD-SGM). Additionally, another characteristic of the model is that the model is interpretable. At present, a large part of the research on pedestrian street-crossing decision-making is concentrated in the field of machine learning. It is well known that a model based on machine learning algorithms is similar to a “black box”, and the model is not interpretable. Once a problem occurs, the source of the problem is difficult to find. This is very dangerous for assisted driving systems or autonomous vehicles [22]. The model proposed in this paper is a computable and interpretable model, which can increase the reliability of the interaction between autonomous vehicles and pedestrians, and improve pedestrian street-crossing safety.

## 2. Materials and Method

### 2.1. Experimental Site

**Figure 1:**
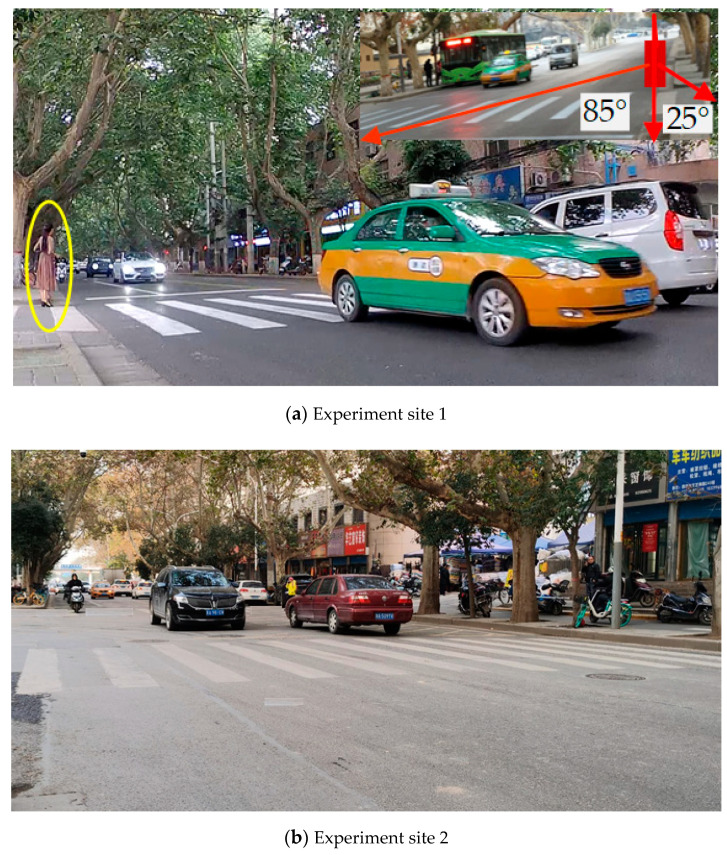
Experimental sites.

Two zebra crossings in Xi’an were selected as the experimental site. The selected zebra crossing without any signal controls is common in Xi’an. The selected road is a two-way four-lane, separated in the middle by a double yellow line, and with each lane being 3 m wide. The selected road is in an urban section, and the traffic flow is mainly composed of private cars, taxis, and buses. Figure 1a,b are photos of different experimental sites.

### 2.2. Apparatus

**Figure 2:**
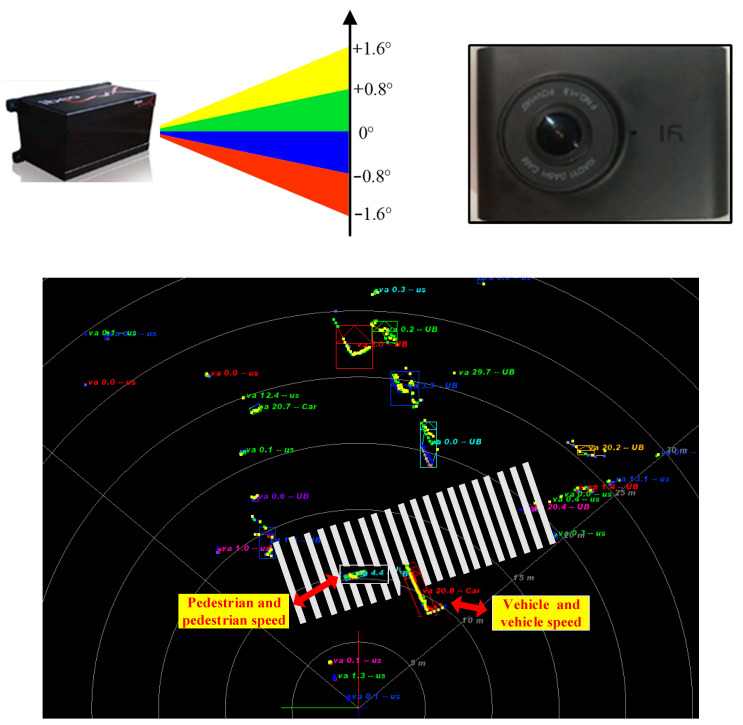
The apparatus and sample lidar data.

A four-layer lidar and high-definition (HD) camera were used to collect data on the pedestrian street-crossing and vehicle movements. The scanning frequency of lidar is 12.5 HZ and its detection range is 0.3 m–200 m. Its vertical viewing angle is 3.2° FOV, and its horizontal viewing angle is 110°. The data collected by lidar includes vehicle speed, pedestrian speed, the distance between the vehicle and the zebra crossing, etc., which can be seen in Figure 2. The resolution of the HD camera is 1920 × 1080. The apparatus was installed on the side of the road, about 15 m away from the zebra crossing and about 0.6 m above the ground. Figure 2 shows the apparatus and sample lidar data.

### 2.3. Data Collection

Data was collected from 7:30 a.m. to 9:00 a.m. and from 5:30 p.m. to 7:00 p.m. every day. In these two time periods, there are many samples of interaction between vehicles and pedestrians. To eliminate the influence of weather on pedestrian decision-making, the data was collected on sunny days only.

During the data selection process, samples without pedestrian-vehicle interaction were eliminated. Similarly, samples with large distances (about 75 m) between pedestrians and vehicles are also considered invalid interaction and are also eliminated [23]. This study focused on two pedestrian street-crossing decisions: crossing and waiting. It should be noted that in this article we only consider the decision-making when pedestrians are about to cross the street, and do not analyze the decision-making process when the pedestrian is in the middle of the street. Two different locations were selected for the experiment. The experiment period is 37 days. The first experimental site is 22 days, 1450 samples are selected, 700 of them are crossing datasets and 750 of them are waiting datasets. The second experimental site is 15 days, 1030 samples are selected, 500 of them are crossing datasets and 530 of them are waiting datasets. Once the data collection was concluded, 2480 samples were selected, 1200 of them crossing the street, and 1280 of them waiting on the curb.

In this work, we have collected the vehicle speed and the distance between the vehicle and the zebra crossing through the lidar. Pedestrian street-crossing decisions (crossing or waiting) can be observed by HD cameras and lidar. Since this study focuses on the vehicle speed at the moment of pedestrian street-crossing decision-making, a certain frame may be occluded and the vehicle speed step may occur during the data selection process. In order to solve the above problems, data interpolation and filtering methods are adopted to reduce errors.

## 3. The Pedestrian Decision-Making Model

### 3.1. Analysis of Related Parameters in Crossing Decision

Generally speaking, pedestrian decision-making on whether or not to cross the zebra crossing depends mainly on whether the external environment poses an immediate threat to their safety (i.e., whether the oncoming vehicles can reduce their speed to zero before reaching the zebra crossing). When the vehicle needs a small deceleration to reduce the speed to zero, the crossing is considered to be safe. When the vehicle needs a large deceleration to reduce the speed to zero or not zero, this is dangerous for pedestrians. Larger vehicle deceleration is described as: suppose the vehicle reduces its speed to 0 before reaching the zebra crossing. When the initial vehicle speed is high and the deceleration distance is short, there will be a large vehicle deceleration. It can be seen, therefore, that the pedestrian decision on whether or not to cross the street is closely related to the safe deceleration of the vehicles. The value of vehicle safety deceleration is the reason for inducing pedestrian street-crossing.

As we all know, the two parameters closely related to vehicle deceleration are vehicle speed and distance between vehicle and zebra crossing. Therefore, when discussing vehicle deceleration, parameters related to vehicle deceleration were also analyzed.

**Figure 3:**
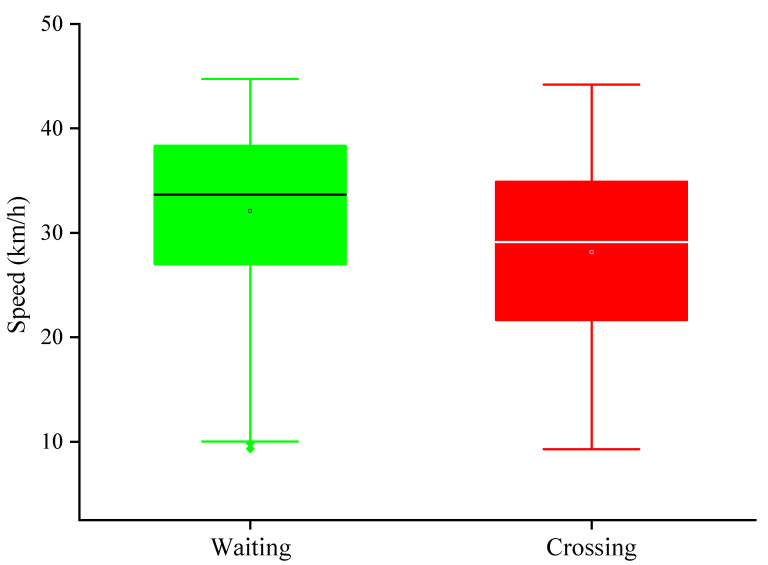
The vehicle speed under different decision-making.

Figure 3 shows the vehicle speeds when pedestrians crossed the street. Statistical analysis indicated that when pedestrians crossed the street, the mean vehicle speed was 28.13 km/h, and when they did not cross, the mean vehicle speed was 32.08 km/h. The one-way analysis of variance (ANOVA) found that the vehicle speed can significantly affect the decision of the pedestrians (F (1, 2478) = 160.61, *p* < 0.05).

**Figure 4:**
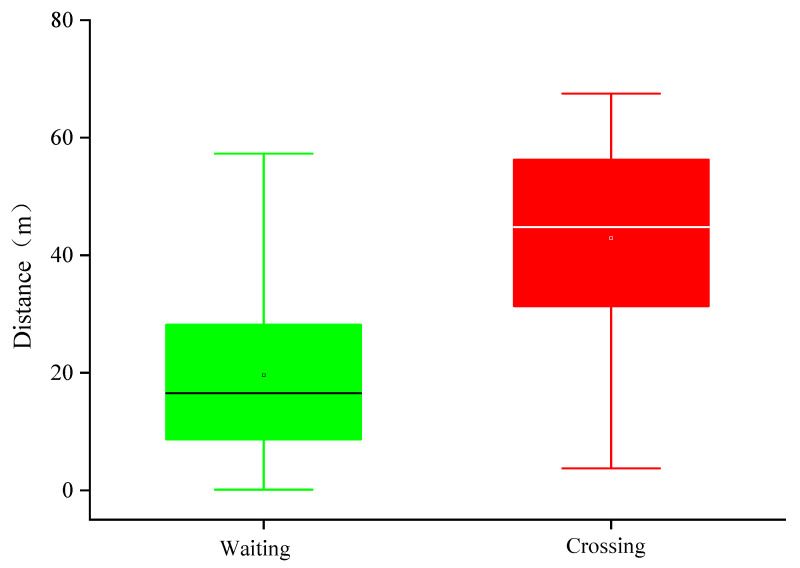
Distance between vehicles and zebra crossings under different decision-making.

According to the conclusion in Reference [23], during the data selection process, samples with large distances (about 75 m) between pedestrians and vehicles are also considered invalid interaction and are also eliminated. Figure 4 shows the distance between the vehicles and the zebra crossings when pedestrians crossing or waiting. Statistical analysis shows that when pedestrians crossed the street, the mean distance between the vehicles and the zebra crossings was 42.52 m. When pedestrians did not cross the street, the mean distance between the vehicles and the zebra crossings was 19.63 m. The one-way ANOVA shows that the vehicle speed can significantly affect the decision of the pedestrians (F (1, 2478) = 1790.65, *p* < 0.05).

**Figure 5:**
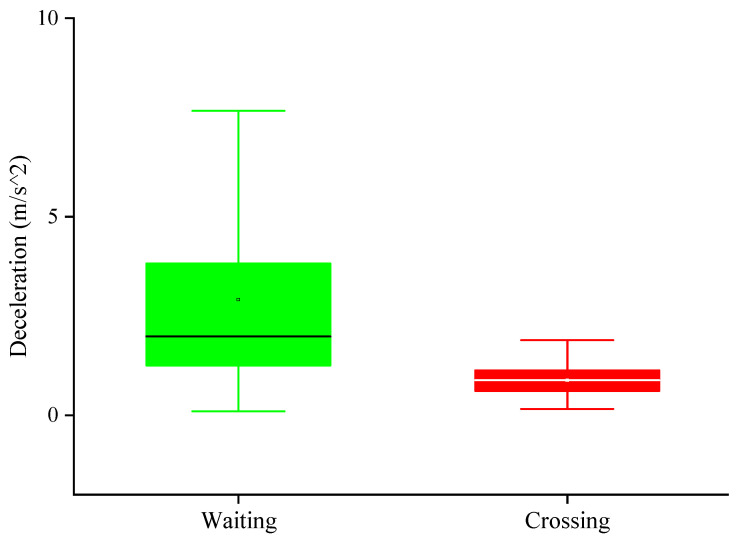
Vehicle deceleration under different decision-making.

Figure 5 shows the vehicle deceleration when pedestrians crossed the street or waited. Statistical analysis shows that the mean value of vehicle deceleration when pedestrians crossed the street was 0.88 $m/s^{2}$. The mean value of vehicle deceleration when pedestrians waited at the curb was 2.91 $m/s^{2}$. It can be seen that when the vehicle deceleration value is relatively small, pedestrians usually choose to cross the road. When the vehicle deceleration value is large, pedestrians usually choose to wait at the curb. The one-way ANOVA found that the vehicle deceleration could significantly affect the decision of pedestrians (F (1, 2478) = 915.35, *p* < 0.05).

### 3.2. Vehicle Deceleration-Safety Gap Model (VD-SGM)

**Figure 6:**
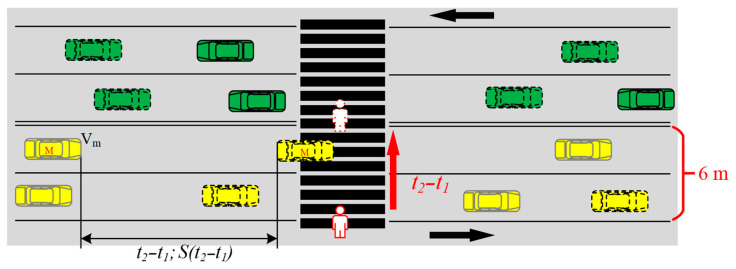
Interactions between the vehicle and pedestrian.

From the results in the previous section, it can be seen that there is a significant difference between the deceleration of the vehicles when pedestrians crossed or waited. This means that it is reasonable to judge pedestrian street-crossing decision by vehicle deceleration. The greater the deceleration required by the vehicle, the more dangerous it is for the pedestrian. Based on the critical safety gap and vehicle deceleration, a VD-SGM was proposed. Figure 6 shows the pedestrian street-crossing process. The modeling principle in this paper is as follows: The time taken for pedestrians from the curb to the middle of the street is $t_{2}-t_{1}$. During this period of time, the smaller the deceleration required for the speed of vehicle M to decrease from $V_{m}$ to 0, the safer the pedestrian is. While The higher the deceleration, the more dangerous the pedestrian is. The model can be presented as follows:

(1) $$a_{m}(t)=f(V_{m}(0),S_{0},SG)$$

(2) $$SG=\{L,V_{1},F\}$$

where $a_{m}\left(t\right)$ is the longitudinal deceleration of vehicle *M*, $V_{m}\left(0\right)$ is the speed of vehicle *M* at the starting of pedestrian street-crossing. $S_{0}$ is the longitudinal distance between the vehicle *M* and the zebra crossing. SG refers to the critical gap, which includes the zebra crossing width *L*, pedestrian street-crossing speed $V_{1}$, and the estimated value of pedestrian loss time *F*, generally 2.5 s [24].

(3) $$SG=\frac{L}{V_{1}}+F$$

(4) $$t_{2}-t_{1}=SG$$

The zebra crossing width *L* is 6 m. According to the investigations into pedestrian street-crossing speed by domestic and foreign scholars, it is mainly concentrated in the 1.0–1.4 m/s range. Out of the desire to make the model safer and taking into consideration the fact that the crossing speed of the elderly is relatively slow, the pedestrian speed was taken as 15% quantile speed, about 1.1 m/s [25,26,27].

From the start time of the crossing $t_{1}$ to the time the pedestrian reaches the central line of the zebra crossing $t_{2}$, the longitudinal displacement of the *M* vehicle is as follows:

(5) $$S_{M}(t)=\int_{0}^{t}\int_{0}^{t}a_{m}(\tau )d\tau dt+V_{m}(0)\times t\begin{array}{ccc} & & t\in [t_{1},t_{2}] \end{array}$$

where $a_{m}$ represents the deceleration of the vehicle and $V_{m}(0)$ represents the initial speed of the vehicle. $S_{M}(t)$ represents the distance traveled by the vehicle in *t* seconds. Pedestrians can be considered safe if the distance traveled by vehicle *M* within the SG is less than the actual distance between the vehicle and the zebra crossing. In other words, the distance traveled by vehicle *M* from the start of crossing time ($t_{1}$) to the moment the pedestrian reaches the middle of the street ($t_{2}$) should be less than the actual distance from the vehicle to the zebra crossing:

(6) $$S_{M}(t)\leq S_{0}\cdot t\in [t_{1},t_{2}]$$

A further arrangement can be obtained as follows:

(7) $$S_{M}(t)=\int_{0}^{t}\int_{0}^{t}a_{m}(\tau )d\tau dt+V{\left(0\right)}_{m}\times t\leq S_{0}\cdot t\in [t_{1},t_{2}]$$

(8) $$t=\frac{V{\left(0\right)}_{m}-V{\left(t\right)}_{m}}{a_{m}}\cdot t\in [t_{1},t_{2}]$$

where V(t) represents the speed of the vehicle after *t* seconds. Substitute Equation (8) into Equation (7) to get Equation (9):

(9) $$S_{M}(t)=\frac{V{\left(0\right)}_{m}^{2}-V{\left(t\right)}_{m}^{2}}{2a_{m}}\leq S_{0}\cdot t\in [t_{1},t_{2}]$$

where V(t) represents the speed of the vehicle after *t* seconds. Since the vehicle speed needs to be reduced to 0 after SG seconds, V(SG) usually takes 0. It can be seen from Equations (7) and (9) that the safety of pedestrian street-crossing depends on the parameters $S_{0}$, $V{\left(0\right)}_{m}$, $a_{m}$, *t*_1_, and *t*_2_.

## 4. Threshold Determination

### 4.1. Signal Detection Theory (SDT)

Signal detection theory (SDT) was first used in 1954 by Tanner and Swets at the University of Michigan to study the human perceptual process. The theory is generally used in studies where the sensations caused by signals and the noise that interferes with signal detection are difficult to distinguish, or where the subjective tendency has a strong influence on the experimental results [28,29,30].

Based on statistical principles, the process of receiving signals from noise interference can be regarded as a statistical judgment process. In the signal detection experiment, four results demonstrate judgments about the presence of the signal: hit, miss, false alarm, and correct rejection. “Hit” refers to an instance when the signal appears (SA), and the subject reports it as “Yes.” It is represented by Y/SA, and the quantity is denoted by f1. This judgment probability is called the conditional probability of a hit, represented by *P* (*H*) or *P* (Y/SA). “Miss” means that when there is a signal, the subject reports it as “none”. The quantity is f2, and it is represented by N/SA. This judgment probability is called the conditional probability of omission, represented by *P* (*M*) or *P* (N/SA). False Alarm refers to the time when there is only noise (NA), but the subject reports “Yes.” The number is expressed as f3 and is expressed as Y/NA. The probability of this determination is called the conditional probability of False Alarm and is expressed as *P* (*FA*) or *P* (Y/NA). Correct rejection means that when noise is present without a signal, the subject reports it as “none.” The quantity is f4, and it is denoted by N/NA. The probability of this determination is called the conditional probability of correct rejection, represented by *P* (*CR*) or *P* (N/NA).

In the experiment of signal detection with background noise, each stimulus state has two reaction possibilities, which can be combined to form an alternative decision matrix. Table 1 presents such a matrix, where *H* and *CR* are correct reactions, *M* and *FA* are wrong reactions, and the probability of each reaction can be expressed as follows:

(10) P(H)+P(M)=1

(11) P(FA)+P(CR)=1

(12) $$P(M)=\frac{f_{2}}{f_{1}+f_{2}}$$

(13) $$P(FA)=\frac{f_{3}}{f_{3}+f_{4}}$$

(14) $$P_{A}=1-\frac{f_{2}+f_{3}}{f_{1}+f_{2}+f_{3}+f_{4}}$$

where *P_A_* presents the accuracy.

### 4.2. Vehicle Deceleration Threshold Selection Based on SDT

When the vehicle needs a small deceleration to reduce the speed to zero, the crossing is considered to be safe. When the vehicle needs a big deceleration to reduce the speed to zero or not zero, this is dangerous for pedestrians. Then, how much vehicle deceleration value can be accepted by pedestrians, and then we use SDT theory to determine. The optimal vehicle deceleration was determined by 1200 crossing samples and 1280 waiting samples. Under the optimal vehicle deceleration condition, the accuracy of the model should be as high as possible, and the false alarm rate and the missing alarm rate should be as low as possible.

**Figure 7:**
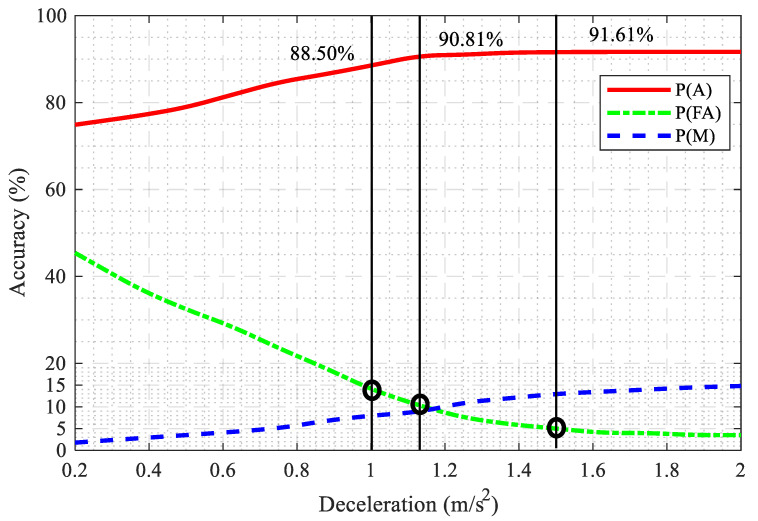
The accuracy, false alarm rate, and miss rates under different thresholds.

The method for determining the vehicle deceleration threshold is as follows:

(1) According to the decision matrix in Table 1, record the state corresponding to each sample when the deceleration threshold takes different values. The status includes hit, false alarm, miss, and correct rejection. The threshold is selected to be calculated in steps of 0.01 s.
(2) Count the false alarm rate, miss rate, and accuracy of all 2480 samples at different thresholds and draw the corresponding curve, as shown in Figure 7.
(3) Analyze the curve to determine the vehicle threshold of the model.

It can be seen that the different false alarm and miss rates are obtained by gradually decreasing the deceleration threshold.

Based on the information in Figure 7, thresholds corresponding to the false alarm rate of 5%, 10%, and 15% were obtained and the corresponding indexes and accuracy rates were calculated. The results are shown in Table 2.

It can be seen from the Table 2 that when the threshold value is 1.50 m/s^2^, the rate of false alarm is 5%, the miss rate is 13.1%, and the accuracy is 91.61%. When the threshold value is 1.13 $m/s^{2}$, the false alarm rate goes up to 10%, the miss rate goes down to 8.83%, and the accuracy goes down to 90.81%. When the threshold value is 1.00 $m/s^{2}$, the false alarm rate rises to 15%, the miss rate falls to 8.17%, and the accuracy lowers to 88.50%. It can be seen that when the deceleration threshold is 1.13 $m/s^{2}$, it is the most appropriate. At this time, the false alarm rate and the missed alarm rate are relatively low. In addition, it can be seen from Figure 7 that when the deceleration is greater than 1.13 $m/s^{2}$, the accuracy rate hardly changes.

When the deceleration threshold is set at 1.13 $m/s^{2}$, the pedestrian street-crossing decision model, namely Equation (9), is adjusted to obtain the following:

(15) $$S_{M}(t)=\frac{V{\left(0\right)}_{m}^{2}}{2.26}$$

To verify the effectiveness of the VD-SGM, it was compared with the Raff model. The Raff method is commonly used in western countries. It is easy and intuitive. The Raff method assumes that the number of rejected gaps is lower than the number of accepted critical gaps. In other words, the intersection point between the rejected and the accepted critical gap curves is the average critical gap value. The Raff value was found to be 3.72 s

**Figure 8:**
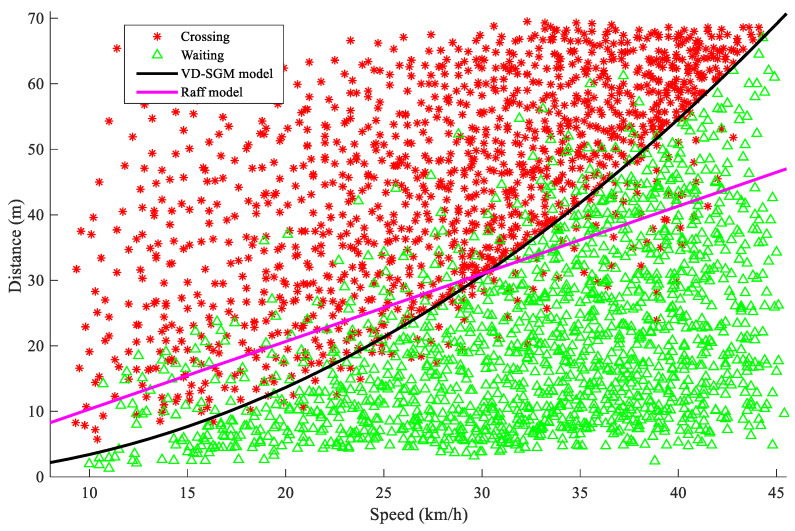
The comparison of the Raff model and the VD-SGM.

The performance of the VD-SGM and the Raff model was evaluated by using the pedestrian waited and crossed data set. The results are shown in Figure 8.

The red points on the scatter plot represent the pedestrians who chose to cross the street, while the green points represent the pedestrians that decided to wait. It can be seen from the Figure 8 that the curve of the model proposed in this paper can accurately show the crossing decision and can effectively differentiate between the crossing and the waiting decisions, while the Raff model curve cannot accurately distinguish the pedestrian street-crossing types.

The two models were further compared and verified based on accuracy rate, miss alarm rate, and false alarm rate, as shown in Table 3. It was found that although the accuracy of the Raff model is high, it is still lower than the vehicle deceleration model proposed in this paper. In addition, the false alarm rate and missed alarm rate of the Raff model are relatively high.

## 5. Discussion and Conclusions

Through naturalistic field experiments, this work establishes a pedestrian street-crossing decision-making model based on vehicle deceleration-safety gap. SDT theory is used to determine vehicle deceleration. The analysis results show that vehicle deceleration is most effective at 1.13 $m/s^{2}$ and the model performs well.

Prior research proposes that TTC, safety gap, and safety distance are important variables of pedestrian street-crossing decision-making. However, these indicators such as TTC and safety gap are impractical because not only do pedestrians not understand these concepts, in many cases they cannot estimate the variables during an encounter with the vehicles (Olszewski, 2020).

One of the major challenges that autonomous vehicles are facing today is driving in urban environments. To achieve this goal, autonomous vehicles need the ability to communicate with other road users and understand their decision-making. This type of interaction is crucial between autonomous vehicles and pedestrians, and a better interaction effect can improve pedestrian safety [31,32]. The most important contribution of this paper is that the VD-SGM more accurately reflects the pedestrian decision-making process about when to cross the street, and the recognition accuracy reached 90.81%. In addition, compared with the pedestrian street-crossing decision based on machine learning algorithms, the model proposed in this paper is computable and interpretable, which increases the reliability of the interaction between pedestrians and autonomous vehicles.

Compared with TTC, the safety gap, and the logistic regression models, the model proposed in this paper is more intuitive. When pedestrians are preparing to cross the street, they may consider whether the vehicle can brake before it crashes into them rather than whether the current TTC or safety gap is large enough. Research by Risto et al. [18], Petzoldt et al. [19], and Zimmermann& Wettach [20] supports the idea that it is reasonable and accurate to judge pedestrian decision about crossing the street based on vehicle deceleration.

The Raff method [3,33]; (Lester, 1988) is easy to calculate, however, pedestrians usually do not pay attention to headway time when crossing a zebra crossing. They may not even understand this concept. In this paper, the VD-SGM is compared with the Raff method. An evaluation of accuracy rate, miss alarm rate, and false alarm rate reveals that the model proposed in this paper has certain advantages.

The VD-SGM is a new human-like model, connecting pedestrian decision-making to vehicle behavior. This model is simple and can more accurately reflect pedestrian street-crossing decisions. The human-like model proposed in this article can be valuable in ensuring self-driving vehicles’ safe pass through zebra crossings and intersections.

Although the computable and interpretable model established in this paper can better reflect pedestrian street-crossing decisions, the model still has some shortcomings, for example, the data during off-peak hours is relatively small and the impact of different weather on pedestrian street- crossing decisions has not been considered. In future work, we will analyze and study the above problems to improve the VD-SGM.

## Figures and Tables

**Table 1:** Alternative decision matrix of pedestrian intention.

	Crossing	Waiting
crossing	Hit *f*1, P(H)	Miss *f*2, P(M)
waiting	False alarm *f*3, P(FA)	Correct rejection *f*4, P(CR)

**Table 2:** Threshold parameters corresponding to the false alarm rate of 5%, 10%, and 15%.

Threshold /(m/s^2^)	False Alarm Rate (%)	Miss Rate (%)	Accuracy Rate (%)
1.50	5	13.00	91.61
1.12	10	8.83	90.81
1.00	15	8.17	88.50

**Table 3:** The summary for the Raff model and deceleration model.

	*P_A_* (%)	*P*(*M*) (%)	*P*(*FA*) (%)
ST1	90.81	8.83	10.00
Raff model	85.68	13.41	15.16
